# Increasing the ethnic diversity of senior leadership within the English National Health Service: using an artificial intelligence approach to evaluate inclusive recruitment strategies in hospital settings

**DOI:** 10.1186/s12960-025-00991-8

**Published:** 2025-05-22

**Authors:** Sarindi Aryasinghe, Catalina Carenzo, Kerri-Ann Barnett, Rabia Khalid, Koya Greenaway-Harvey, Colleen Sherlock, Louise Clark, Kevin Croft, Tim Orchard, Erik Mayer

**Affiliations:** 1 iCARE Secure Data Environment, NIHR Imperial Biomedical Research Centre, Imperial College Healthcare NHS Trust, London, UK; 2 Faculty of Medicine, Department of Surgery and Cancer, Imperial College London, London, UK; 3 Office of the Chief Executive and People and Organisational Development Directorate, Imperial College Healthcare NHS Trust, London, UK

**Keywords:** Equality, Diversity, Inclusion, Ethnicity, Recruitment, Discrimination, Health leadership, Natural language processing, Artificial intelligence, Human resource analytics

## Abstract

**Background:**

The English National Health Service (NHS) strives for a fair, diverse, and inclusive workplace, but Black and Minority Ethnic (BME) representation in senior leadership roles remains limited. To address this, a large multi-hospital acute NHS Trust introduced an inclusive recruitment programme, requiring ethnically and gender diverse interview panels and a letter to the Chief Executive Officer (CEO) explaining hiring manager’s candidate choice. This generated large amount of valuable structured and free-text data, but manual analysis to derive actionable insights is challenging, limiting efforts to evaluate and improve such equality, diversity, and inclusion (EDI) recruitment initiatives.

**Methods:**

Using this routinely collected recruitment data from the programme between September 2021 to January 2024, we used natural language processing artificial intelligence techniques, triangulated with secondary data analysis, to evaluate the programme’s effectiveness in increasing the number of BME appointees to senior leadership roles. Multivariate logistic regression identified recruitment factors that influence the odds of BME candidates applying, being shortlisted or offered a role compared to white candidates. Topic and sentiment analysis revealed thematic trends and tone of candidate assessments, stratified by hiring manager and candidate characteristics. Normalised average interview scores were also compared by job grades and candidate characteristics.

**Results:**

The requirement for hiring managers to write a letter to the CEO explaining recruitment decisions raised the odds of a BME candidate being offered a role by 1.7 times [95% CI 1.2–2.3] compared to white candidates. However, white candidates still had higher overall odds of being offered senior roles. BME candidates scored lower in interviews, with BME women twice as likely (*p* < 0.05) to receive negative assessments compared to white women.

**Conclusions:**

The Letter to the CEO component of the inclusive recruitment programme increased BME representation in senior leadership roles, but inequities still persist in the recruitment process, reflecting national NHS recruitment trends. While the initiative marks progress, further strategies are needed to ensure equitable recruitment, career development, and retention. Artificial intelligence tools, such as natural language processing, provide effective methods to evaluate and enhance EDI recruitment initiatives by analysing routinely collected recruitment data to identify areas for improvement and establish best practices.

**Supplementary Information:**

The online version contains supplementary material available at 10.1186/s12960-025-00991-8.

## Background

The National Health Service (NHS) is one of the world’s largest employers, with its constitution highlighting the importance of a fair and non-discriminatory workplace for all staff [1]. Despite having one of the most ethnically diverse workforces in the public sector [2], ethnic inequalities in NHS senior leadership representation persists [3]. These disparities are influenced by individuals’ protected characteristics and sociodemographic status, affecting employment opportunities, career progression, and treatment at work [4]. National policies, including the 2020 NHS People Plan and 2023 NHS Equality, Diversity, and Inclusion Improvement Plan, urge NHS employers to prioritise diversity by fostering inclusion and implementing fair recruitment and workforce management strategies to increase diversity of under-represented groups [5–7].

Nationally, 26.4% of NHS staff are from Black and Minority Ethnic (BME) backgrounds. However, in 76% of NHS Trusts, white applicants are more likely than BME applicants to secure senior roles, with NHS staff engagement surveys demonstrating that BME staff have unequal workplace experiences compared to their white colleagues [4, 8]. Only 11.2% of very senior manager positions are filled by BME staff, and 39% of staff from Black ethnic backgrounds believe their Trust offers equal career progression opportunities, irrespective of their gender [8]. Despite this, existing evidence on recruitment bias, career progression and retention focus primarily on gender rather than other protected characteristics such as ethnicity [4], considering that 76% of the NHS national workforce are women [9].

A diverse healthcare workforce benefits patients and organisations by addressing disparities and enhancing care for under-served communities. For example, demographic concordance between patients and providers improves communication, decision-making, patient adherence to care plans, and earlier uptake of preventative care [10–12]. Fairer treatment of staff also increases the likelihood that patients have a positive care experience [13]. Organisational diversity can also influence employee satisfaction within the workplace [14]. From a human resources perspective, it broadens the talent pool to enhance workforce sustainability while boosting productivity through greater efficiency and innovation [15].

As a result, many workplaces have implemented diversity initiatives to improve recruitment, career progression, and retention, though causality remains difficult to establish due to workplace complexities and data limitations [4]. Unstructured free-text data, which represents a substantial proportion of human resources information, can be time-consuming and labour-intensive to derive meaningful insights on these diversity initiatives [16]. Artificial intelligence (AI) methods, particularly natural language processing (NLP), can efficiently reveal hidden patterns in words and phrases used such as themes or sentiment that may be overlooked if manually analysed [17].

However, evidence on the use of AI approaches to explore effectiveness and operationalisation of workforce diversity recruitment strategies remains under-explored [18]. Therefore, this study aims to demonstrate the use of AI using NLP to assess the effectiveness of an inclusive recruitment programme to increase the ethnic diversity of senior leadership within an English NHS Trust.

## Methods

### Study design

This study analysed routine data from the inclusive recruitment programme in a large acute NHS Trust to assess its impact on hiring outcomes for BME and white candidates across application, shortlisting, and offer stages, with gender stratification for an intersectional lens where possible [19]. The research team held monthly meetings with the Trust’s People and Organisational Development (OD) team to guide study design, data processing, analysis, and review emerging results and programmatic implications.

### Study setting

Imperial College Healthcare NHS Trust (ICHT) is an acute teaching hospital trust in North West London. One of the largest Trusts in England, it serves over 1.3 million patients annually with 16,000 employees across five hospitals [20]. 70% of the Trust’s workforce are women, with similar proportions occupying senior roles. When looking at ethnicity, 62% of its non-clinical and 61% of its clinical workforce are from a BME background but are under-represented in leadership positions [21]. As an anchor organisation, the Trust prioritises addressing ethnic health inequities and ensuring equality in employment and progression, with a key goal of its Equality, Diversity, and Inclusion (EDI) strategy being to create a workforce that reflects the communities it serves [22]. To achieve this objective, an inclusive recruitment programme was implemented in September 2021 to increase the ethnicity and gender diversity in senior leadership roles, defined as Agenda for Change (AfC) band 7 roles and above [23]. This banding range was chosen as Band 7 is the first level for leading a team or service in clinical roles, equating to a service manager in non-clinical NHS roles. The programme includes two components: (i) implementation of ethnicity and gender compliant interview panels and (ii) having hiring managers, who are responsible for the hiring decision, write a letter to the Trust Chief Executive Officer (CEO) to explain their candidate choice. The letter to the CEO became a mandatory requirement in June 2022.

#### Recruitment process at the Trust

Recruitment at Imperial College Healthcare NHS Trust begins with applicants exploring vacancies on the Trust and NHS Job websites, with some vacancies only internally advertised. After reviewing job descriptions, applicants create accounts on the NHS Jobs online platform [24] to complete the online application forms (including personal details and key questions related to the job position) and track application progress. Post-deadline, hiring managers and interview panels complete a blinded shortlisting process against the person specification. Shortlisted candidates are then invited for an interview. Afterwards, a gender and ethnicity-diverse panel deliberates to select the most qualified candidate, documenting the process, rationale for appointable candidates, and feedback details for unsuccessful candidates in a letter to the CEO. A copy of the letter is emailed to the CEO, the People and OD team, and the hiring manager themselves. Once the letter is submitted, successful candidates receive a verbal offer followed by a formal offer letter detailing employment terms and conditions. Random spotchecks of the letters are also done by the CEO, with hiring managers occasionally invited for an informal discussion to review the letter's content.

### Data sources

Routinely collected data as part of the Trust’s inclusive recruitment programme was used for this analysis. All interview campaigns across the Trust were included during three time periods: (i) between July 2020 to June 2021 to establish a baseline before the implementation of the inclusive recruitment programme; (ii) between September 2021 to June 2022, where both components of the inclusive recruitment programme were a recommendation; and (iii) after July 2022 and until January 2024, where the letter to the CEO became a mandatory requirement. Data before July 2020 was unavailable for analysis, as inclusive recruitment was not considered an organisational priority at that time. Structured data included the AfC Banding of the role (grade), number of applications, shortlisting, offers, and panel characteristics. Letters to the CEO provided unstructured data on panel details, candidate assessments, and interview outcomes (Additional file 1).

### Data cleansing and processing

A unique table consolidated panel composition and candidate characteristics at each recruitment stage. Data from letters to the CEO was extracted using Optical Character Recognition (OCR) in Python, retrieving details on panel composition, candidates, and interview assessments. Letters with inconsistent structures or formatted text (e.g., strikethrough, highlighted) that hindered OCR recognition were discarded. Each letter provided data on panel members (role, seniority, ethnicity, gender) and candidates (ethnicity, internal/external, unnormalised interview score). NLP techniques extracted hiring managers’ gender which was primarily manually coded by themselves as binary (women/men). Candidate gender was presented using pronouns used by hiring manager to describe the candidate (e.g., "he/his/Mr" or "she/her/Miss/Ms"), which were extracted and identified using the Python library "gender_guessor", with manual validation for accuracy. Consolidated data was anonymised using an approved (by the Trust Data Protection Office and Caldicott Guardian) de-identification algorithm in the iCARE secure data environment, with follow-up manual inspection by the iCARE team prior to analysis. Free-text answers were standardised into predefined categories and converted using one-hot encoding where relevant, represented by 1 s and 0 s. For example, the data point "Interview panel compliant" was standardised, with "Yes" or "N" as 1 (compliant) and 0 (non-compliant) (Table 1).

**Table 1:** Representation of the raw data made available through the inclusive recruitment programme and how it was transformed into a format for analysis

Data item	Description	Examples of raw data	Final format of processed data for analysis
Baseline workforce data (structured)			
Overall workforce	Overall Trust population numbers, stratified by the Office of National Statistics (ONS) aggregated ethnicity categories	Black/African/Caribbean/ Black British; White; Asian/Asian British; other Ethnic Group; Mixed/Multiple ethnic groups	No change
New hires	Overall new starters numbers, stratified by ONS Ethnicity Categories	Black/African/Caribbean/ Black British; White; Asian/Asian British; other Ethnic Group; Mixed/Multiple ethnic groups	No change
Inclusive recruitment programme routine data (structured)			
Job ref	Job description reference number for the role	202-R4E-209	No change
Division	Trust division of the role	Division A, Division B, Division C, etc	Standardised division names, given that different acronyms were used for the divisions
Job title	Title of the job	Clinical Specialist	No change
Grade	Agenda for Change (AfC) banding of role	7, 8a, 8b, band 8c, 8d, 9	7, 8a, 8b, 8c, 8d, 9
Interview Date	Date of the interview	17 Sep 2023, 17 September 2023	dd-mm-yyyy (17–09-2023)
Vacancy advertised Internally or externally	Indicates whether the vacancy was advertised internally or externally	Internal, Y, E	Internally-advertised vacancy coded as 1 Externally-advertised vacancy coded as 0
N of applicants	Number of applications received for the role	10	No change
N of applicants BME	Number of Black and Minority ethnic candidates that applied for the role	8	No change
N shortlisted	Total number of candidates that were shortlisted	3	No change
N shortlisted BME	Number of Black and Minority ethnic candidates that were shortlisted	2	No change
N Offers	Number of offers made for the role—once conditional offer has been given	1	No change
N offered BME	Number of BME candidates that were offered the role	1	No change
Interview panel Gender compliant	Indicates whether the interview panel was gender compliant (i.e. there was a woman on the interview panel)	No, compliant, Y, N	Gender compliant panel coded as 1 Gender non-compliant panel coded as 0
Interview panel BME compliant	Indicates whether the interview panel was ethnicity compliant (i.e. there was a BME individual on the interview panel)	No, compliant, Y, N	Ethnicity compliant panel coded as 1 Ethnicity non-compliant panel coded as 0
N successful candidates Internal	Number of successful internal candidates	0	No change
N successful candidates External	Number of successful external candidates	1	No change
Interview Outcome form received	Indicates whether the interview outcome form stating successful candidates was received by the People and OD team	Y, 1, No	Interview outcome received coded as 1 Interview outcome not received coded as 0
Letter to the CEO received	Indicates whether the Letter to the CEO was received by the People and OD team	Yes, No, 0	Letter to the CEO received coded as 1 Letter to the CEO received coded as 0
Letters to the CEO (unstructured)			
Job title and reference number	Job title description and reference number	Clinical Specialist 202 -R4e -209	Job reference: 202-R4E-209
Interview date	Date of the interview	17 Sep 2023, 17 September 2023	17–09-2023
Role	Role of the member at the interview panel	Nurse	No change
Gender	Gender of the interview panel member	F, M, Woman, Female, Man	Woman panel member coded as 1 Man panel member coded as 0
Panel member ethnicity	Ethnicity of the interview panel member	B, BME, W, Asian	BME panel member coded as 1 White panel member coded as 0
Band/grade	Band/grade of the interview panel member	7, 8a, 8b, band 8c, 8d, 9	7, 8a, 8b, 8c, 8d, 9
Internal/external	Indicates whether the interview panel member was internal (working for the NHS) or external	I, Ext, 1	Internal interview panel member coded as 1 External interview panel member coded as 0
Involved in shortlisting?	Indicates whether the interview panel member was involved in shortlisting	Yes, N, 1	Involved in shortlisting coded as 1 Not involved in shortlisting coded 0
Inclusive recruitment trained?	Indicates whether the interview panel member has taken the inclusive recruitment training offered by the Trust	Yes, N, 1, not sure	Inclusive recruitment trained coded as 1 Not inclusive recruitment trained coded as 0
Candidate name	Name of the candidate	Mary Smith, John Doe	Female candidate coded as 1 Male candidate coded as 0
Internal/ external candidate	Indicates whether the candidate interviewed was internal (already working for the NHS) or external	I, E, Internal, External, Yes, No	Internal candidate coded as 1 External candidate coded as 0
Candidate ethnicity	Indicates the ethnicity of the candidate	B, BME, W, White	BME candidate coded as 1 White candidate coded as 0
Summary of assessment	Anonymised summary of assessment of the candidate being interviewed agreed by the interview panel to the candidate being interviewed	“The candidate proved knowledge on..”	"candidate proved knowledge”
Interview score (out of total)	Score given by the interview panel to the candidate being interviewed	23/30, 50/70	76.67/100, 71.4/100
Appointable	Indicates whether the candidate interviewed was appointable	Yes, No, Not appointed, appointed	Appointed candidate coded as 1 Non-appointed candidate coded as 0

### Data triangulation

Data harmonisation and triangulation were crucial for developing a comprehensive data set, integrating information from both spreadsheets and letters for each interview campaign. The unique job identifier, present in most documents, served as the primary key for aligning data. To manage inconsistencies in job identifiers due to manual entry, Python’s Regex library was used for robust matching, followed by manual comparisons from both sources ensured data integrity. Discrepancies such as missing values were resolved through cross-referencing and stakeholder discussions. Certain interview campaign characteristics, such as job type (e.g., allied health professionals), were excluded from the analysis due to challenges in data triangulation between the manually compiled data sources.

### Data analysis

We employed a three-step approach to data analysis, which included regular stakeholder input from People and OD team and the Trust’s EDI Committee to ensure accurate and actionable outputs to improve the programme.

#### Analysis of structured data

Using descriptive analysis, we analysed trends in BME new starters before and after the programme, examining candidate ethnicity and gender at application, shortlisting and offer stages, stratified by AfC Banding (Band 7, 8a, 8b, 8c, 8d and 9) and type of vacancy (internal or externally advertised). We also compared the current number of BME new starters to the ethnic make-up of the Trust workforce, stratified by ethnicity using the Office of National Statistics ethnicity categories [25].

To assess the odds of a white candidate applying, being shortlisted, or offered a role compared to a BME candidate, we conducted multivariate logistic regression analyses reviewing the data from September 2021 to March 2023, covering both the recommendation and mandatory phases of the inclusive recruitment programme. This enabled the assessment of which factors influenced recruitment outcomes, such as AfC Banding, gender and ethnicity compliance of the interview panel, and completion of a letter to the CEO. Multivariate logistic regression was most appropriate as it enabled us to statistically determine the influence of multiple factors at the same time, allowing us to assess the unique contributions of each factor after controlling for the effects of others [26]. Data beyond March 2023 was excluded as all subsequent interview campaigns included a letter and a compliant interview panel. This ensured enough variation in these factors, preventing the model from wrongly assuming certain features had no effect due to insufficient contrasting examples [27, 28].

#### Natural language processing (NLP) of the letters to the CEO

To gain a more comprehensive understanding of the recruitment process and the impact of the programme, we utilised NLP to analyse the thematic trends using an inductive approach [16] in the letters to the CEO, triangulated where possible with the structured data for granularity. By combining this qualitative data with relevant structured data, we explored how factors like the hiring panel composition and the characteristics of the role affect which candidates are shortlisted and appointed to roles within the Trust.

##### Text pre-processing

To enhance the quality and reliability of the analysis, all text from letters to the CEO was standardised to minimise structural variations using Natural Language Toolkit (NLTK), a Python library for NLP. This involved techniques such as stemming and lemmatisation (reducing words to their root or base form) and removing stop words such as common words and pre-defined phrases from letters to the CEO templates (e.g., “the”, “and”, “is”, “The following examples of their knowledge, skills, and experience were evidenced"). These steps reduced the vocabulary size to only the most relevant words in the text, making the data more manageable, informative and focused, thereby improving the efficiency of the NLP algorithm.

##### Calculating impact of panel members on candidate interview scores

Using data about the interview panel extracted through NLP, we assessed the impact of having a compliant panel on candidate interview scores. To allow fair comparisons across interviews, each candidate’s score was normalised to a 0 to 100 scale with the maximum score per interview as 100 and scaling accordingly. The scores were then stratified by gender, ethnicity and banding. Interview campaigns from Band 8 and 9 were combined, because the small sample size of Band 9 roles alone would not be suitable for statistical testing. To assess the significance of mean score differences among these stratified candidate groups, we conducted *Z* tests and Welch’s *t* tests. Before these analyses, normality was checked with the Shapiro–Wilk test, and variance equality with Levene's test.

##### Sentiment analysis

Sentiment analysis was used to gauge the emotional tone in candidate assessments by candidate and hiring manager ethnicity and gender [29]. These were categorised as positive, neutral, or negative using TextBlob, an open-sourced and widely used Python NLP library trained on a large data set of labelled customer feedback and social media text samples. 10% random sample of the NLP outputs were assessed manually to understand if the correct sentiment had been assigned. If a consistent pattern of errors were identified, then the NLP model was adjusted, and the comments reprocessed using the corrected approach. Two-proportion *Z* tests was used to assess the significance of differences in feedback with negative sentiment by candidate gender and ethnicity characteristics.

##### Topic analysis of candidate strengths and weaknesses commentaries

We conducted topic analysis on free-text fields to identify themes in the candidates' strengths and weaknesses written by hiring managers in the letters to the CEO. Using an unsupervised machine learning approach, we developed an algorithm to group similar word patterns into topics (e.g., strengths: “benefit from”, “good at”; weaknesses: “not enough”, “lack of”) [30, 31]. Text was transformed numerically using term frequency–inverse document frequency (TF–IDF). Both latent dirichlet allocation (LDA) and non-negative matrix factorization (NMF) from Scikit-learn were explored to extract topics from the data; with the main objective being to develop human interpretable and deterministic topics [31]. NMF yielded the most coherent results, based on the authors’ knowledge and expertise in EDI and workforce development. Similar to the sentiment analysis, 10% of the candidate assessments were manually reviewed by the data scientist for accuracy. The resulting topics were then stratified by candidate and hiring manager characteristics.

## Results

In total, 1716 interview campaigns were analysed between September 2021 and January 2024, spanning both the recommended and mandatory phase of the inclusive recruitment programme, with 792 (46.2%) vacancies being internally advertised. 1615 interview campaigns had an accompanying letter to the CEO. Through data triangulation with the structured recruitment data, 1070 letters to the CEO were included for NLP (Table 2).

**Table 2:** Number of interview campaigns and Letters to the CEO analysed

Division	No. of interview campaigns	No. of applications	No. of shortlisted candidates	No. of candidates offered a role	Letters to CEO included in analysis	Letters to CEO triangulated with structured data
Division A	369	5452	1037	343	273	204
Division B	423	3858	1002	407	514	281
Division C	431	3555	1017	428	366	266
Division D	404	2786	671	365	392	249
Division E	89	922	332	85	70	70
Total	1716	16,573	4059	1628	1615	1070

### Overall candidate characteristics

In the analysed interview campaigns, 64.7% of all applicants (*n* = 10,726) were from a BME background. Among those shortlisted, 59.3% (*n* = 4058) were BME candidates. Of those who received an offer, approximately half (*n* = 820) were from a BME background. Table 3 provides a further breakdown by ethnicity and gender of a subset of these candidates at the shortlisting and offer stage using the 1070 triangulated letters to the CEO.

**Table 3:** Number of candidates shortlisted and offered in triangulated letters to the CEO

Candidate characteristics	No. of shortlisted candidates	No. of candidates offered the role
White women	1079	602
BME women	653	289
White men	713	284
BME men	984	329
Total	4059	1504

### Odds ratios of BME candidates at each recruitment stage

At the application and shortlisting stages, BME candidates have higher odds than white candidates of applying or being shortlisted for Band 7 and 8 roles, with the odds decreasing with role seniority. When a vacancy is advertised internally within the organisation, a BME candidate has 30% less odds [95% confidence interval (CI) 0.1–0.9] than a White candidate of being shortlisted for the role. At the offer stage, BME candidates have significantly less odds of being offered a role across Bands 7 to 9 than their white counterparts. However, when a letter to the CEO is written by the hiring manager as part of the recruitment process, a BME candidate has 1.7 times [95% CI 1.2–2.3] the odds of being offered a role than a white candidate. A BME candidate also has 1.9 times [95% CI 0.4–10.0] the odds than a white candidate of being offered a role if the interview panel is gender and ethnicity compliant, but this result was not statistically significant (Table 4).

**Table 4:** Factors affecting the odds of BME candidates compared to white candidates at each recruitment stage

Factors	OR and 95% confidence interval of BME candidate at each recruitment stage compared to white candidates		
	Application	Shortlisted for interview	Offer
Band 7.0	6.6 [5.0–8.3]*	5.3 [4.2–6.7]*	0.6 [0.4–0.9]*
Band 8.0a	5.4 [3.7–7.7]*	5.4 [3.9–7.7]*	0.5 [0.3–0.7]*
Band 8.0b	5.0 [2.9–8.3]*	5.1 [3.0–8.3]*	0.5 [0.3–0.9]*
Band 8.0c	5.3 [2.3–12.5]*	2.2 [1.2–4.2]*	0.2 [0.1–0.4]*
Band 8.0d	3.7 [1.4–10.0]*	1.8 [0.8–4.2]	0.3 [0.1–0.8]*
Band 9.0	1.3 [0.5–3.3]	0.6 [0.2–1.6]	0.1 [0.04–0.5]*
Internally advertised vacancy	0.9 [0.7–1.3]	0.7 [0.1–0.9]*	1.3 [1.0–1.7]*
Letter to CEO written	Not applicable		1.7 [1.2–2.3]*
Interview panel gender and ethnicity compliant			1.9 [0.4–10.0]
Interview panel only gender compliant			0.5 [0.1–2.2]
Interview panel only ethnicity compliant			0.9 [0.4–2.0]

^*^p < *0.05* = *the odds ratio was statistically significant*

### Impact of gender and ethnicity compliant panel on interview scores

Since the implementation of the inclusive recruitment programme, 96% of all interview panels were gender and ethnicity compliant. After normalising all interview scores from 0 to 100 to enable comparisons, analysis showed that regardless of whether there was a gender and ethnicity compliant panel, the average normalised interview score was 5 percentage points lower for BME women than white women for band 7 roles (*z* = − 2.03, *p* < 0.05). Within Band 8 and 9 roles, the average 6 percentage points was lower (*t*(502)  = − 2.12, *p* < 0.05) for BME men than white men, with Levene’s test indicating unequal variances (*F* = 5.39, *p* < 0.05) (Table 5).

**Table 5:** Normalised average interview scores (from 0 to 100) stratified by AfC banding and candidate characteristics

Candidate characteristics	Normalised average interview score (Band 7)	Normalised average interview score (Band 8 and 9)
White women	62 (*p* < 0.05)	61
BME women	57 (*p* < 0.05)	57
White men	57	59 (*p* < 0.05)
BME men	54	53 (*p* < 0.05)

### Sentiment analysis

The sentiment of candidate assessments was analysed by candidate and hiring manager ethnicity and gender. Phrases such as “Demonstrated good understanding of…”, “considered to be good addition to the team”, “excellent knowledge” were classified as having a positive sentiment. Examples of negatively worded phrases were “Very poor interview”, “Clearly was not able to demonstrate knowledge”, or “Lacked confidence”. Examples of neutrally worded phrases include “Ability to communicate”, “Didn’t meet all criteria”, “Understood job requirements”.

**Fig. 1:**
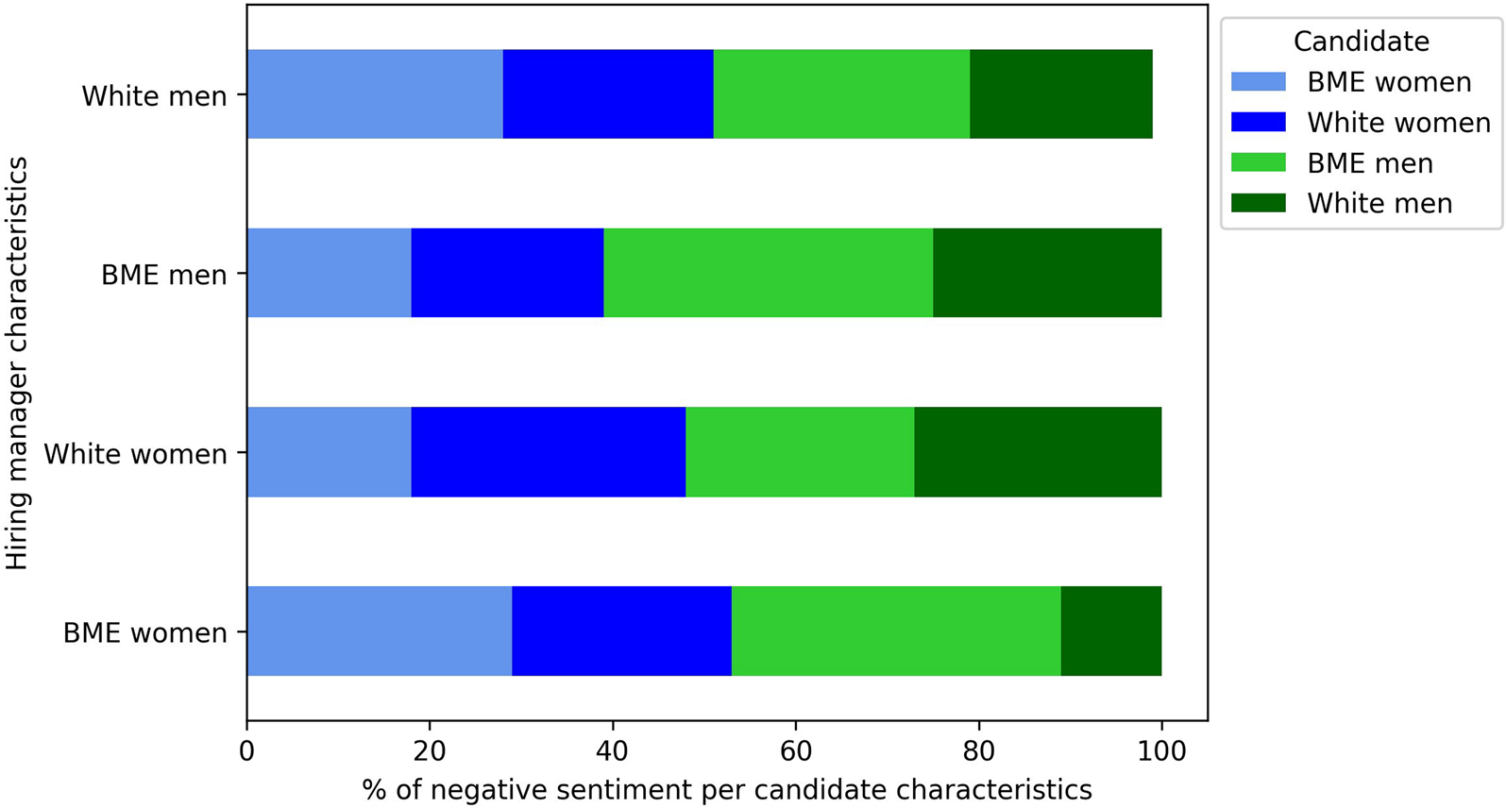
Distribution of negative sentiment, stratified by candidate and hiring manager characteristic

Overall, 88% of the candidate assessments had a positive sentiment, while 5% were negative. Of these negative sentiments, BME women candidates were 1.6 times (*p* < 0.05) more likely to receive negative feedback than white candidates, with no significant differences in neutral assessments. BME women hiring managers also gave less negative feedback to white candidates than BME men. BME men hiring managers provided more negative feedback to men candidates, regardless of ethnicity, while white men hiring managers gave more negative feedback to BME candidates, irrespective of gender (Fig. 1).

#### Topic analysis

Topic analysis using an unsupervised machine learning approach identified key themes in free-text descriptions of candidate strengths and weaknesses. 79% of the candidate assessments described candidate strengths, with the following four topics: understanding of the role (“understood requirements for the role”); skills and knowledge (“knowledge of operational ability”); previous experience (“good experience in acute Trust”); and interview performance (“was very clear and concise in their answers”). 65% of the assessments included candidate weaknesses across four themes: people and management skills (“Unable to show management skills”); meeting requirements (“Knowledge on research was not fully evidence”); quality of answers (“Could have used better examples to answer questions”); and further support needed (“I would recommend further training on…”).

#### Topic analysis stratified by candidate ethnicity and gender

**Fig. 2:**
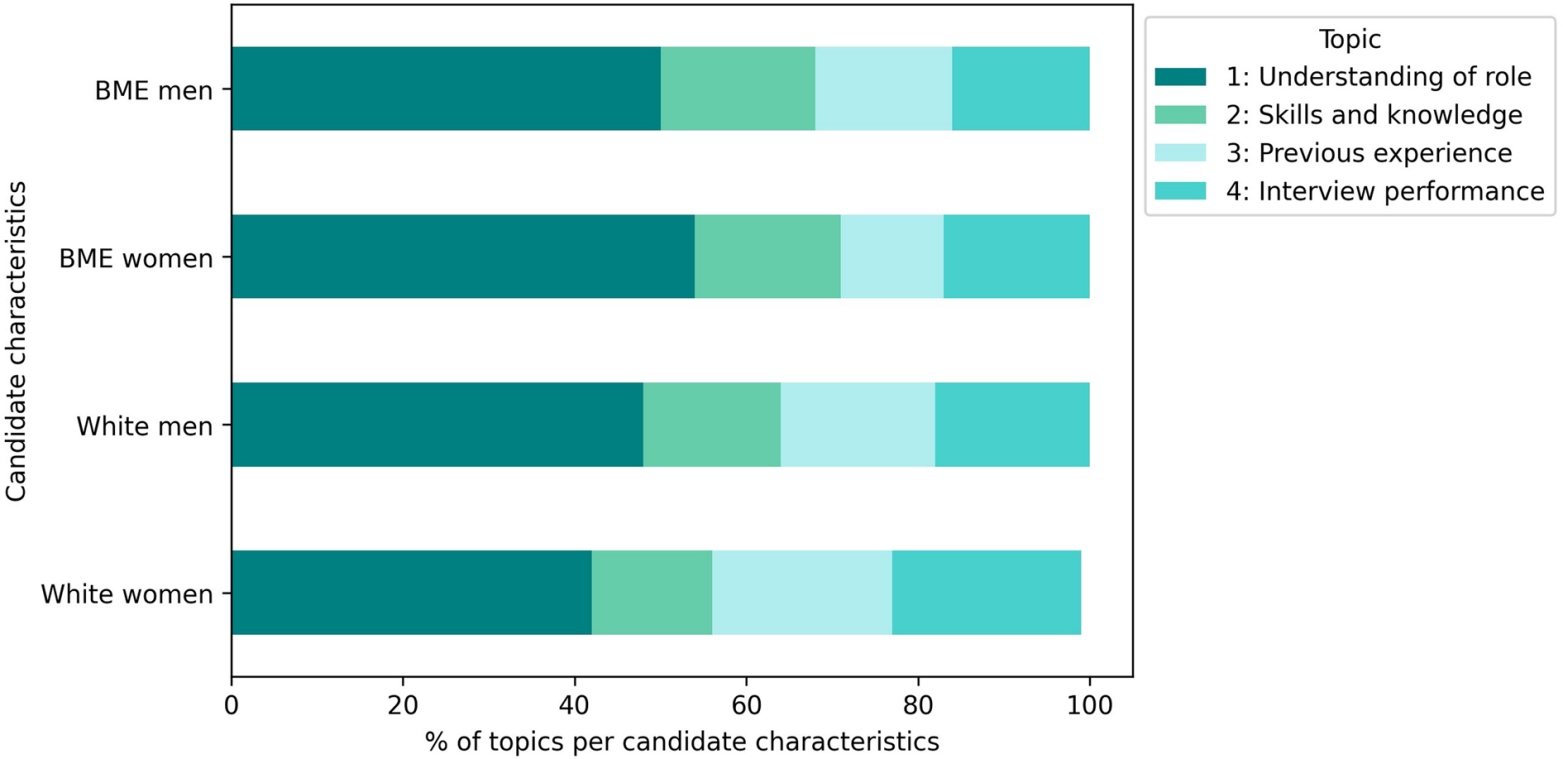
Strengths topic analysis stratified by candidate ethnicity and gender

**Fig. 3:**
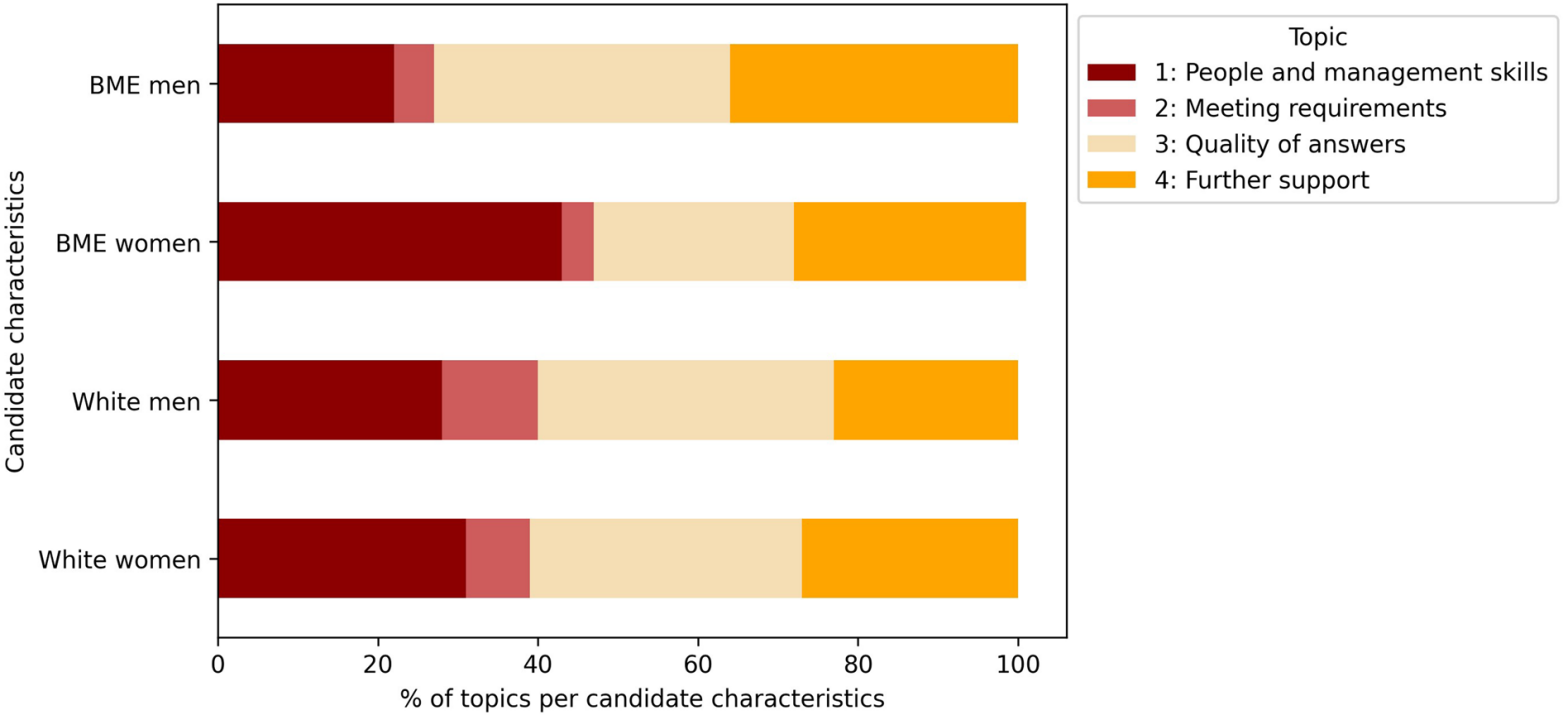
Weaknesses topic analysis stratified by candidate ethnicity and gender

Overall, all candidates received positive evaluations for their understanding of the role. However, when the topic analysis was stratified by ethnicity and gender, the specific strengths varied. Interview performance was particularly highlighted for white women, while prior experience was less frequently noted for BME women (Fig. 2). For weaknesses, white candidates, particularly men, were more commonly seen as not meeting role requirements, whereas BME women were more frequently identified as having weaknesses in people and management skills (Fig. 3).

#### Topic analysis stratified by hiring manager characteristics

**Fig. 4:**
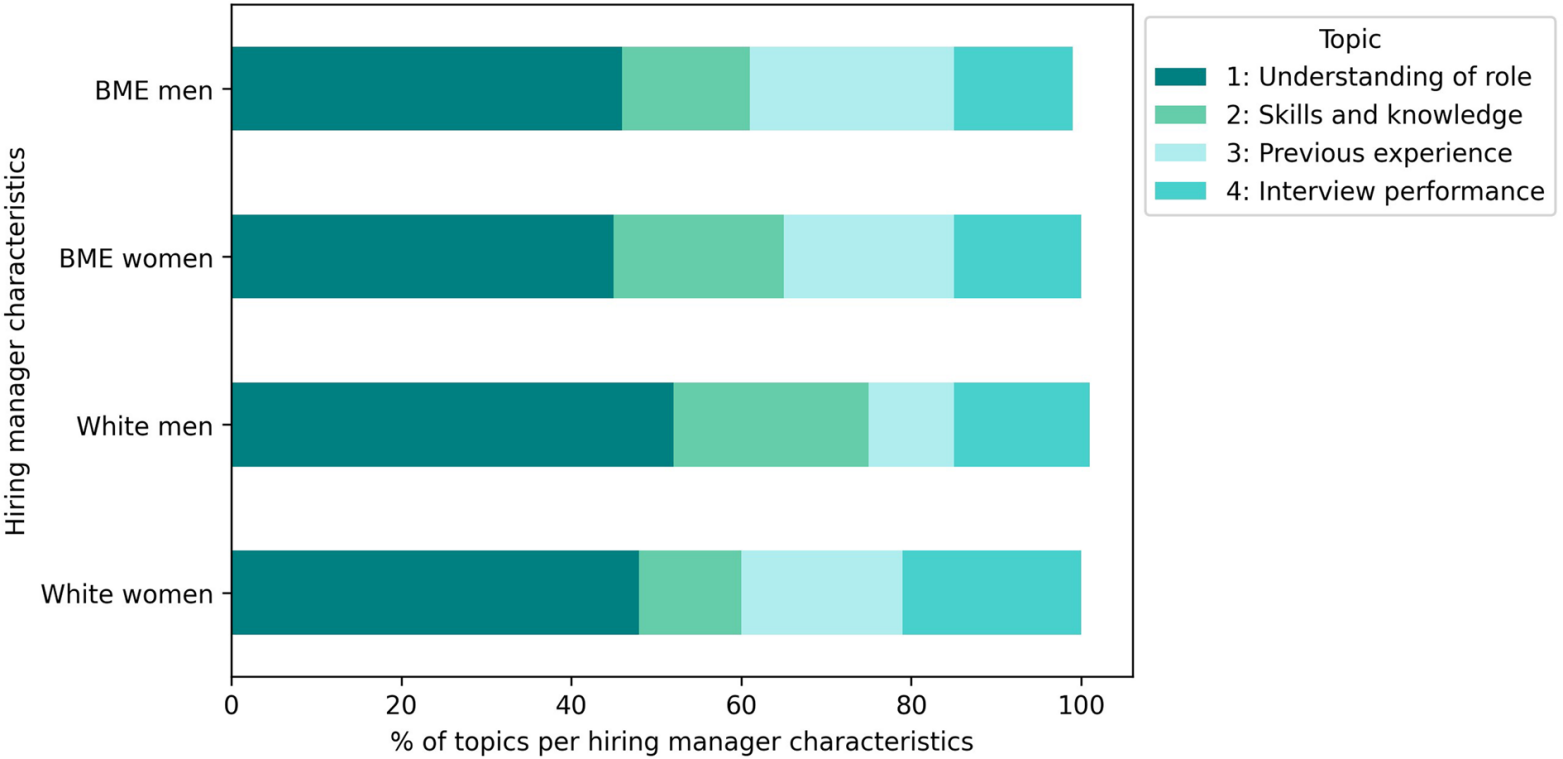
Strengths topic analysis stratified by hiring manager ethnicity and gender

When analysing the distribution of topics by hiring manager characteristics, a candidate’s understanding of the role was the most frequently cited strength by all managers. BME men and women hiring managers showed similar patterns, with BME men hiring managers emphasising candidates' previous experience more than other hiring managers. In contrast, white men hiring managers prioritised skills and knowledge, while white women hiring managers more frequently highlighted interview performance as a key strength (Fig. 4).

**Fig. 5:**
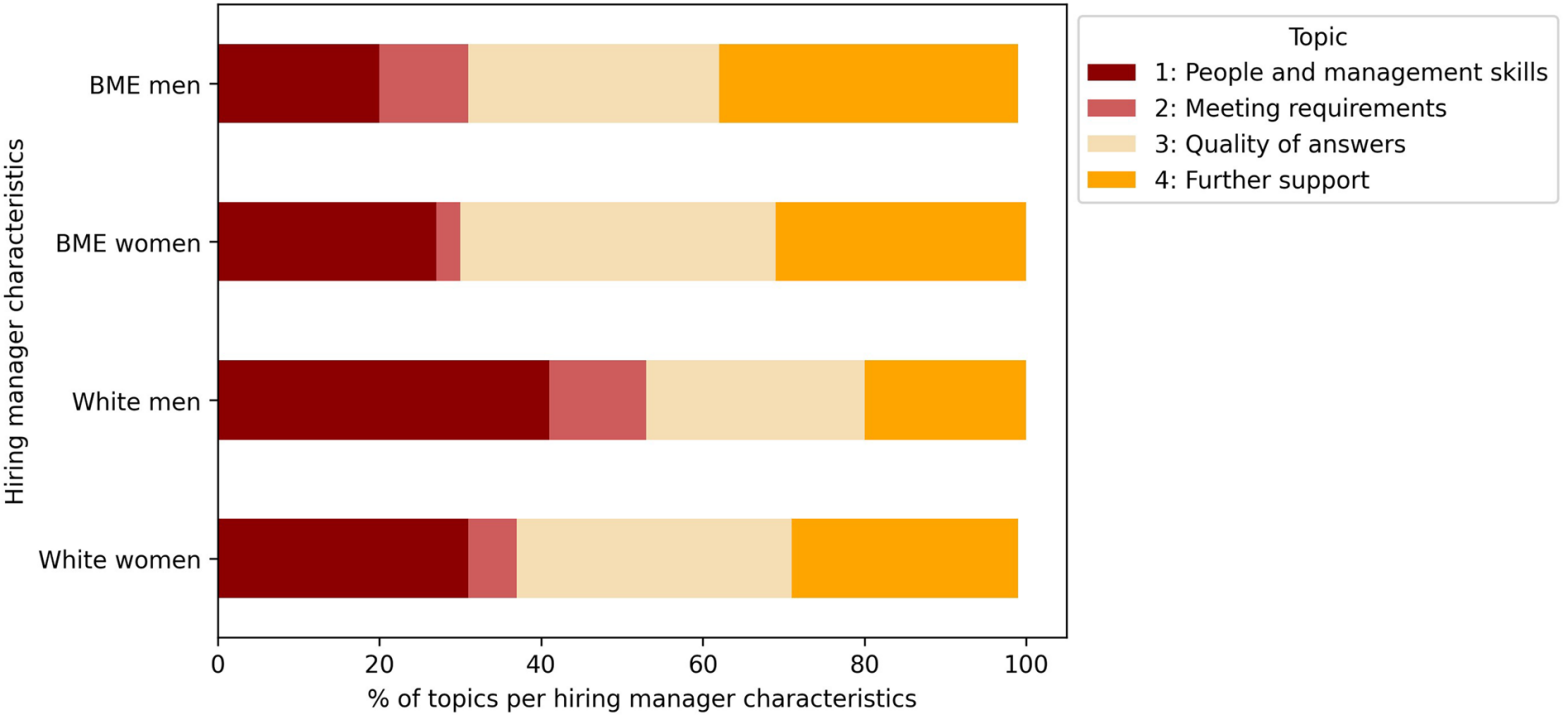
Weaknesses topic analysis stratified by candidate ethnicity and gender

For weaknesses, white men hiring managers tended to focus less on recommending additional support and more on deficiencies in candidates' people and management skills. White women hiring managers placed equal emphasis on people and management skills as well as the quality of candidates’ answers. BME men hiring managers were more inclined to suggest additional support, while BME women hiring managers concentrated on poor answer quality to interview questions, with less focus on candidates meeting the role requirements (Fig. 5).

## Discussion

Our analysis of the inclusive recruitment programme data assessed its impact on enhancing diversity in senior leadership roles within a large acute NHS Trust. Requiring hiring managers to submit a letter to the CEO explaining their recruitment rationale nearly doubled the odds of a BME candidate being offered a senior leadership role compared to a white candidate. However, white candidates still have higher odds of receiving job offers, with BME candidates scoring lower in interviews than white candidates, highlighting the need for further measures to address recruitment bias and continue embedding the inclusive recruitment programme.

Our findings align with national NHS recruitment trends, where white candidates are more likely than BME candidates to be shortlisted and appointed to a role [32]. Our analysis demonstrated BME candidates also have reduced odds of being shortlisted for internally advertised vacancies, and this disadvantage appears to deepen with each promotion [4], mirroring findings from Warmington et al. [33] that BME careers often stagnate, leading to lateral moves and additional qualifications to improve prospects; this non-linear progression is, however, sometimes perceived negatively by selection panels. Although ICHT's panels complete blinded shortlisting, these factors may still influence decision-making.

While most interview panels met gender and ethnicity requirements, BME women candidates received more negative assessments than their white counterparts. Notably, BME women hiring managers gave fewer negative assessments to white men candidates, suggesting that panel dynamics, including member seniority and decision-making, as well as the format of the assessments require further investigation. Therefore, racial bias and preferences can be present regardless of an interview panel’s ethnic makeup. It is also recognised that certain assessment types such as unstructured interviews and personal statements are less likely to promote diversity, while structured interviews and situational judgement tests offer greater reliability, validity, and support for diverse candidates [34].

Nonetheless, the mandatory letter to the CEO initiative proved effective in increasing the odds of a BME candidate being offered a senior leadership role. As the CEO reviews a random selection of letters, this signals to employees that workforce diversity is an organisational priority, reinforcing accountability [35–37]. Further support from divisional recruitment managers and the Trust’s EDI committee also enables consistent reinforcement and sustainability of such diversity initiatives over time [36, 38]. Despite letters to the CEO increasing the odds of BME candidate appointments, white candidates continue to have increased odds of being shortlisted and hired, indicating that no single intervention can drive inclusive recruitment [39]. Managers must still be equipped with knowledge about diversity, discrimination, and bias, and understand their impact on recruitment, engagement, and retention [40]. Job descriptions need to include inclusive wording to encourage applications from diverse candidates [41]. Ongoing targeted training, transparent promotion, reward processes, and dedicated support for employees with protected characteristics—such as access to professional development, peer networks, and clear career progression opportunities—will further contribute to a work culture of inclusion and fairness [32].

### Next steps and recommendations

The analysis identified opportunities to strengthen the inclusive recruitment programme. To reduce the drop-off of BME candidates from application to shortlisting, clear guidance on shortlisting procedures—including roles and numbers—should enhance objectivity. Given that BME women often receive negative assessments, training for hiring managers on delivering constructive feedback is crucial. In addition, correlating these findings with the organisational staff engagement survey data could offer deeper insights into employees' experiences regarding career progression and development support. Integrating data from the NHS national application system (TRAC) would improve accuracy and detail of candidate protected characteristics (e.g., having ethnicity and gender breakdowns according to Office of National Statistics categories), allowing for more robust intersectional analysis. Standardised, disaggregated data on panel members and hiring managers at each stage would also help to pinpoint areas for improvement. These programmatic improvements, alongside the availability of more detailed data, offer a pathway to further strengthen the inclusive recruitment programme so that inequities in recruitment outcomes are minimised.

### Strengths and limitations

The strength of this study lies in its combined use of structured and unstructured recruitment data, providing nuanced insights into inclusive recruitment strategies within the NHS and optimising human resources data that is otherwise challenging to analyse due to resource limitations. Using an intersectional lens, we examined how ethnicity and gender of both candidates and hiring managers influenced recruitment outcomes at application, shortlisting, and offer stages, enabling the People and OD team to create targeted solutions. This framework also supports future studies of other protected characteristics, such as disability, age, and sexual orientation. In addition, collaboration between the research team and the People and OD team strengthened the relevance and programmatic accuracy of our findings [42]. Their domain expertise and input helped ensure explainability of the results for staff through simple but meaningful visualisations, as well as supported the interpretation of the NLP topic analysis outputs [17, 43].

However, establishing "what works" in addressing discrimination in recruitment and evaluating effective interventions is complex [4]. This study assessed an inclusive recruitment programme using pre- and post-implementation data, and we could not control for divisional or team-level implementation, as these were undocumented. For example, interview selection processes can vary by job role and team, with some candidates undergoing additional stages like presentations. In addition, limited recruitment data disaggregated by ethnicity and gender in the years before the programme—due to not historically being an organisational mandate—restricted our ability to assess its full effects on influencing BME progression into the senior roles. Identifying the full pool of applicants is also challenging due to exclusions from visa restrictions or qualifications, which particularly affect BME candidates and may contribute to their high application rates and subsequent reduced odds of being shortlisted. Certain NHS professions, such as allied health professionals, lack ethnic diversity, limiting the pool of diverse candidates for senior promotion [44]. Therefore, a two-pronged approach is needed: identifying diverse candidates at junior levels and addressing recruitment bias to support progression. This study focuses on the latter and aims to provide evidence on an acute Trust’s strategy to improve ethnic diversity in senior roles.

In addition, the data quality issues in the letters to the CEO and recruitment data limited insights into the hiring process, such as further detailed analysis by job roles. Incomplete shortlisting panel data and free-text descriptions of candidate and hiring manager characteristics made it difficult to analyse how panel’s make-up influenced which candidates were invited for interviews. For example, candidate’s gender was not explicitly collected but was referenced by hiring managers in their assessments in a binary way. Furthermore, while odds ratios showed associations between recruitment outcomes and demographics, causation remains unclear, necessitating further contextual analysis. Finally, granularity and robustness of the topic and sentiment analysis of candidate assessments could benefit from a larger set of letters to the CEO.

## Conclusion

Overall, the inclusive recruitment programme has had a positive impact on increasing the odds of BME candidates being appointed to a senior leadership role at the Trust, but the overall trends in recruitment still mirror national trends within the NHS of inequities in hiring outcomes between BME and white candidates at shortlisting and offering stage. Despite this, the completion of a letter to the CEO increases the odds of a BME candidate being offered a senior leadership role. Therefore, this study suggests that the inclusive recruitment programme has been effective in increasing the diversity of the workforce at senior leadership levels within the NHS, but holistic approaches should still be taken to support the recruitment, development, and retention of staff.

## Supplementary Information

Additional file 1.

## Data Availability

The data sets generated and analysed in the current study are not publicly available as they contain confidential organisational recruitment data. External sharing is not covered by the institution’s service evaluation agreement. No datasets were generated or analysed during the current study.

## Abbreviations

AfC: Agenda for change
AI: Artificial intelligence
BME: Black and minority ethnic
CEO: Chief executive officer
EDI: Equality, diversity, and inclusion
ICHT: Imperial College Healthcare NHS Trust
LDA: Latent Dirichlet Allocation
NHS: National Health Service
NLP: Natural language processing
NLTK: Natural language toolkit
NMF: Non-negative matrix factorisation
OCR: Optical character recognition
OD: Organisational development
TD–IDF: Term frequency–inverse document frequency
