# Supplementary material for: Increasing the ethnic diversity of senior leadership within the English National Health Service: using an artificial intelligence approach to evaluate inclusive recruitment strategies in hospital settings
Source: Hum Resour Health. 2025 May 22;23:24. doi: 10.1186/s12960-025-00991-8 (PMC12096476; doi:10.1186/s12960-025-00991-8)
Supplement: Supplementary file 1 — Additional file 1. [file 12960_2025_991_MOESM1_ESM.docx]

DEPARTMENT/DIRECTORATE

SITE

ADDRESS

London

POSTCODE

DATE

Chief Executive Officer

Address

London

Postcode

Dear [CEO name]

**RE: Interview outcome from the Chair of the Interview Panel**

| **Directorate/department** | **Job title** | **Trac job Reference Number** | **Interview date** |
| --- | --- | --- | --- |
| Department |  | 290 - | Interview date |

In line with the Trust’s commitment to improve transparency and fairness in the recruitment process, I am writing to provide an overview of the interview and selection process for the above post so that you can be assured that the process we have undertaken has been truly inclusive.

Overall observations and comments

The ethnicity and gender of the panel was / was not compliant with our policy to have gender and ethnicity diverse interview panels. IF NOT, PLEASE PROVIDE RATIONALE.

Following the interview, the panel discussed and agreed on the most suitable candidate. There was / was not consensus on the preferred candidate(s). In order to reach a decision, the panel INSERT ACTION TAKEN.

As panel chair, I have provided feedback to all candidates. DELETE IF NOT APPROPRIATE; Those candidates that were internal (XX), will be provided with support for their development from MANAGER NAME to aid future applications within the Trust and have been provided with the link and encouraged to sign up to the Managing People Well programme and signposted to the Career Focus webpage. Of the unsuccessful internal candidates, there were XX who are from an ethnic minority. In addition to the above, these candidates have been provided with the contact details for our learning and development team on XXX@nhs.net to access additional career development and support.

Should you have any questions or concerns on this recruitment episode, please contact me [XXX@nhs.net](mailto:XXX@nhs.net), [phone number]

Yours sincerely

NAME
JOB TITLE

Please complete the rest of the report below and send this to your Recruitment Officer and to [CEO’s email] within 2 working days of the interview date.

**Report from the Chair of the Interview Panel**

Advertisement - The post was advertised on **NHS Jobs, Trac** and INSERT ANY OTHER MEDIA. The advert was published for XX days.

Selection/shortlisting process

| **Role** | **Name and job title** | **Gender** | **Ethnicity**  **Note 1* | **Grade/Band** | **Internal/ external** |
| --- | --- | --- | --- | --- | --- |
| **Lead shortlister** | Name and job title | M/F/non-binary | B/W/O | X | Internal / External |
| **Shortlister** | Name and job title | M/F/non-binary | B/W/O | X | Internal / External |
| **Shortlister** | Name and job title | M/F/non-binary | B/W/O | X | Internal / External |

X candidates were shortlisted and X attended the selection process. The selection process consisted of interview/ presentation/ online test/ assessment/ stakeholder event/ other.

Selection Panel members - The interview panel consisted of the following people.

| **Role** | **Name and job title** | **Gender** | **Ethnicity**  **Note 1* | **Grade/Band** | **Internal/ external** | **Involved in shortlisting?** | **Inclusive recruitment trained?** |
| --- | --- | --- | --- | --- | --- | --- | --- |
| **Chair** | Name and job title | M/F/non-binary | B/W/O | X | Internal / External | Yes / No | Yes / No |
| **Panel member** | Name and job title | M/F/non-binary | B/W/O | X | Internal / External | Yes / No | Yes / No |
| **Panel member** | Name and job title | M/F/non-binary | B/W/O | X | Internal / External | Yes / No | Yes / No |
| **Panel member** | Name and job title | M/F/non-binary | B/W/O | X | Internal / External | Yes / No | Yes / No |

Note 1: Ethnicity * B = Black, Asian and Minority Ethnic, W = White, O = Other / Not stated

Candidates Interviewed

Interviews were held on INSERT DATE and XX candidates were seen on the day. The selection panel agreed that the following assessments and that X candidate(s) were appointable. A summary of the candidates is as follows;

| **Candidate First name and Surname** | **TRAC ID** | **Appointable** | **Interview score (out of total)** | **Summary of assessment – areas of strengths and weaknesses** | **Date feedback provided** | **Internal / external** | **Ethnicity**  **Note 1* |
| --- | --- | --- | --- | --- | --- | --- | --- |
| First name Surname | TRAC ID | YES / NO | XX / XX | The following examples of their knowledge, skills and experience were evidenced:  XXXX  The following elements of the person specification were not evidenced, or were not as strong as the successful candidate:  XXX  The reasons this candidate was considered appointable / not appointable was:  XXX | DATE | Internal / External | B/W/O |
| First name Surname | TRAC ID | YES / NO | XX / XX | The following examples of their knowledge, skills and experience were evidenced:  XXXX  The following elements of the person specification were not evidenced, or were not as strong as the successful candidate:  XXX  The reasons this candidate was considered appointable / not appointable was:  XXX | DATE | Internal / External | B/W/O |
| First name Surname | TRAC ID | YES / NO | XX / XX | The following examples of their knowledge, skills and experience were evidenced:  XXXX  The following elements of the person specification were not evidenced, or were not as strong as the successful candidate:  XXX  The reasons this candidate was considered appointable / not appointable was:  XXX | DATE | Internal / External | B/W/O |

Note 1: Ethnicity *

B = Black, Asian and Minority Ethnic

W = White

O = Other / Not stated

**Recruitment team to populate ethnicity of candidates: white / BAME / not stated**
